# Antioxidant Effects of Bovine Lactoferrin on Dexamethasone-Induced Hypertension in Rat

**DOI:** 10.1155/2014/943523

**Published:** 2014-01-22

**Authors:** Leila Safaeian, Hadi Zabolian

**Affiliations:** Department of Pharmacology and Toxicology and Isfahan Pharmaceutical Sciences Research Center, School of Pharmacy and Pharmaceutical Sciences, Isfahan University of Medical Sciences, Isfahan 81746-73461, Iran

## Abstract

Dexamethasone- (Dex-) induced hypertension is associated with enhanced oxidative stress. Lactoferrin (LF) is an iron-binding glycoprotein with antihypertensive properties. In this study, we investigated the effect of chronic administration of LF on oxidative stress and hypertension upon Dex administration. Male Wistar rats were treated by Dex (30 **μ**g/kg/day subcutaneously) or saline for 14 days. Oral bovine LF (30, 100, 300 mg/kg) was given from day 8 to 14 in a reversal study. In a prevention study, rats received 4 days of LF treatment followed by Dex and continued during the test period. Systolic blood pressure (SBP) was measured using tail-cuff method. Thymus weight was used as a marker of glucocorticoid activity. Plasma hydrogen peroxide (H_2_O_2_) concentration and ferric reducing antioxidant power (FRAP) value were determined. Dexamethasone significantly increased SBP and plasma H_2_O_2_ level and decreased thymus and body weights. LF lowered (*P* < 0.01) and dose dependently prevented (*P* < 0.001) Dex-induced hypertension. LF prevented body weight loss and significantly reduced the elevated plasma H_2_O_2_ and increased FRAP values. Chronic administration of LF strongly reduced the blood pressure and production of ROS and improved antioxidant capacity in Dex-induced hypertension, suggesting the role of inhibition of oxidative stress as another mechanism of antihypertensive action of LF.

## 1. Introduction

Chronic administration of glucocorticoid, especially at supraphysiological doses, leads to elevated systolic blood pressure, in man and animals [1]. Increased vascular sensitivity to glucocorticoids has been also demonstrated in patients with essential hypertension [2]. Dexamethasone- (Dex-) induced hypertension is associated with decreased antioxidant levels, enhanced oxidative stress and reduced nitric oxide (NO) level [3, 4]. Overproduction of reactive oxygen species (ROS) occurs in this model of hypertension like many other forms of hypertension. Interaction between ROS and NO leads to reduced NO availability and vasoconstriction [5]. An increasing body of evidence has shown the role of antioxidants in preventing increased oxidative stress and blood pressure in Dex-induced hypertension [6–8].

Lactoferrin (LF) is a multifunctional iron-binding glycoprotein. Different biological fluids especially milk and also neutrophilic granules contain LF [9]. LF receptors are found in various cell types such as lymphocytes, platelets, macrophages, dopaminergic neurons, megacaryocytes, and endothelial cells. Some of these receptors are involved in LF uptake. In the cerebral endothelial cells, LF is transported through a receptor-mediated process without any intraendothelial degradation [10].

LF acts as the first-line defense against bacterial, fungal, and viral infections [11]. It has anti-inflammatory effect and its production is increased during inflammatory circumstances [12].

LF is a regulator of organ morphogenesis and promotes wound healing and bone growth. It has enzymatic activities in some reactions and also some anticancer activities [13].

LF has been identified as an antioxidant protein with ability to increase antioxidant capacity and decrease ROS formation [14–16]. Recently, some studies have demonstrated antihypertensive effects of this food-derived peptide and its hydrolysate in normal and spontaneously hypertensive rats (SHR) [17, 18]. The NO-dependent vasodilation, inhibition of angiotensin I-converting enzyme (ACE) activity, and inhibition of endothelin-converting enzyme (ECE) activity have been suggested as the mechanisms responsible for antihypertensive effects of LF [19–21].

The present study aimed to evaluate blood pressure lowering effects of chronic LF in Dex-induced hypertension and to determine the role of inhibition of oxidative stress as another probable mechanism of action of LF in prevention of hypertension.

## 2. Materials and Methods

### 2.1. Experimental Animals

Male Wistar rats (200–230 g) were obtained from the animal house of the School of Pharmacy and Pharmaceutical Sciences, Isfahan University of Medical Sciences, Iran. They were kept in standard laboratory conditions with free access to food and water. Rats were acclimated to the laboratory conditions for at least one week before the experiments. Animals were weighed on alternate days. All experiments were performed according to the internationally accepted guidelines for laboratory animal use and care.

### 2.2. Chemicals

Bovine lactoferrin (Sigma-Aldrich Co., USA), dexamethasone (Raha Pharmaceutical Co., Iran) and captopril (Tehran Darou, Iran), were used in this study. Plasma lipid hydroperoxides measurement and ferric reducing antioxidant power (FRAP) assay were performed using standard assay kits (East Sage Research Co., Iran).

### 2.3. Experimental Protocol

Animals were treated by subcutaneous (s.c.) administration of Dex (30 *μ*g/kg/day) for induction of hypertension as the Dex control group or saline (1 mL/kg) as the saline control group for 14 days [22]. In a prevention study, rats received oral LF (30, 100, 300 mg/kg) or captopril treatment (40 mg/kg; a known angiotensin-converting enzyme inhibitor, as the positive control) using an intragastric tube for 4 days followed by Dex administration and continued during the test period (14 days). In the reversal study, LF or captopril was administered from day 8 to day 14. Six animals were used in each group. At the end of the experiment, all groups of animals were sacrificed under ether anesthesia. Blood samples were collected into heparinized tubes and plasma was separated. The thymus gland was also isolated.

### 2.4. Measurement of Systolic Blood Pressure

Systolic blood pressure (SBP) was measured every week using noninvasive tail-cuff method (AD Instrument PowerLab Data Acquisition System, Australia) in conscious rats. The animals were individually restrained in a clear acrylic restrainer at an ambient temperature of 37-38°C for 15 min. Five blood pressure values were recorded for each rat and the average of them was taken as the SBP. To assure the reliability of the measurements, a training period of one week was established before the experiment time for adaptation of rats to the method.

### 2.5. Measurement of Thymus Weight

The thymus gland was weighed and expressed as milligrams per 100 g body weight. The thymus weightwas used as a marker of glucocorticoid activity [23].

### 2.6. Measurement of Plasma Hydrogen Peroxide Concentration

Plasma hydrogen peroxide (H_2_O_2_) concentration was measured based on the ferrous ion oxidation by xylenol orange (FOX) assay using a standard assay kit according to the manufacturer's protocol [24]. Briefly, FOX reagent was mixed with plasma samples and incubated for 30 min in 37°C. The absorbance of colored samples was read at 540 nm with ELISA/spectrophotometric reader. Plasma H_2_O_2_ concentration was calculated against the hydrogen peroxide standard curve absorption values.

### 2.7. Plasma Ferric Reducing Antioxidant Power (FRAP) Assay

The antioxidant capacity of plasma samples was determined by FRAP assay based on the reduction of ferric-tripyridyltriazine complex to ferrous form using a standard assay kit [25]. Briefly, the FRAP reagent was added to plasma samples and incubated for 40 min in 40°C. The absorbance of colored samples was read at 570 nm using an ELISA/spectrophotometric reader. The values were calculated against the standard curve of FeSO_4_.7H_2_O concentration and absorption values were expressed as micromole of ferrous ion equivalents per liter.

### 2.8. Statistical Analysis

The data were expressed as the mean ± SEM. Statistical analysis was made by one way analysis of variance (ANOVA) followed by Tukey post hoc test using SPSS 16.0 software and GraphPad-Prism 5 software. *P* value < 0.05 was considered statistically significant.

## 3. Results

### 3.1. Effect of Lactoferrin on Blood Pressure

**Figure 1:**
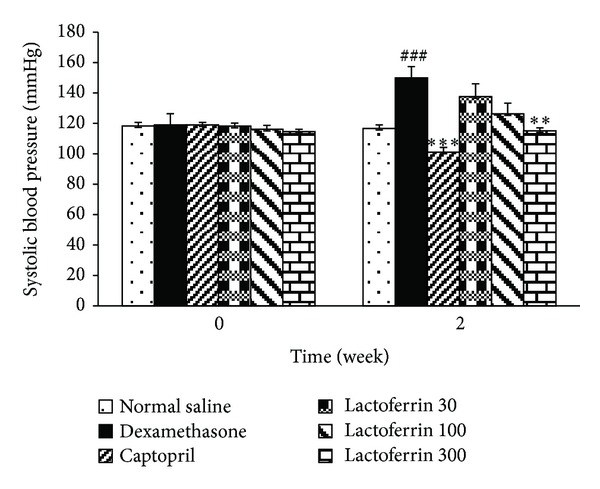
Effects of oral lactoferrin (30–300 mg/kg) and captopril (40 mg/kg) on systolic blood pressure on Dex-induced hypertension in reversal groups. Values are means ± SEM for six rats. As compared to Dex control group, **: *P* < 0.01, and ***: *P* < 0.001. As compared to saline control group, ^###^: *P* < 0.001.

**Figure 2:**
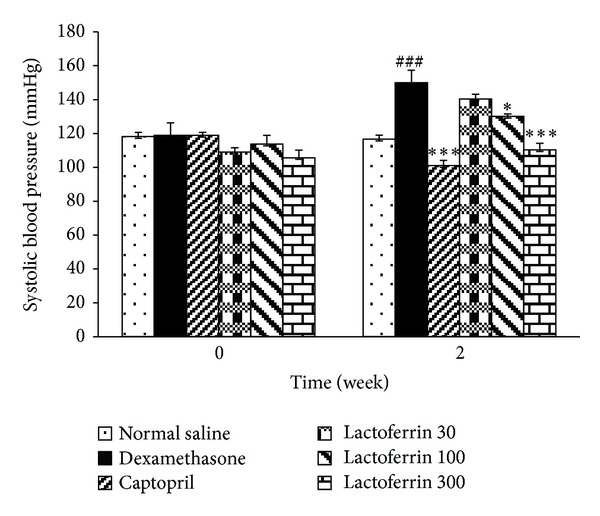
Effects of oral lactoferrin (30–300 mg/kg) and captopril (40 mg/kg) on systolic blood pressure on Dex-induced hypertension in prevention groups. Values are means ± SEM for six rats. As compared to Dex control group, *: *P* < 0.05, ***: *P* < 0.001. As compared to saline control group, ^###^: *P* < 0.001.

Dexamethasone significantly increased SBP from 118.9 ± 4.7 to 140.44 ± 10.3 mmHg on day 7 (*P* < 0.01) and to 150 ± 7.4 mmHg on day 14 (*P* < 0.001) in comparison with saline control group (116.6 ± 2.4 mmHg). The oral administration of LF (30–300 mg/kg) lowered and dose dependently prevented Dex-induced hypertension in reversal and prevention studies (Figures 1 and 2).

### 3.2. Effect of Lactoferrin on Body Weight

**Figure 3:**
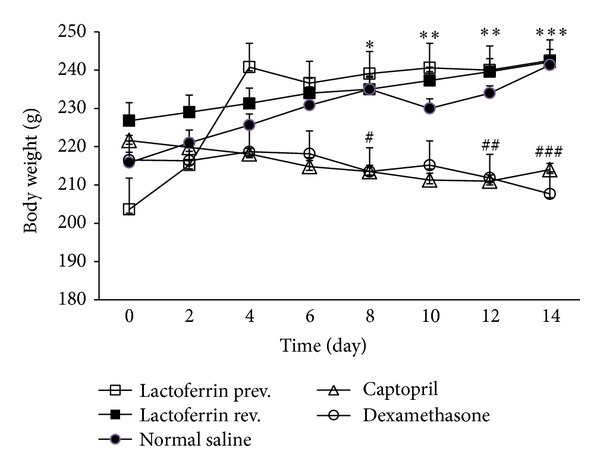
Effects of oral lactoferrin (300 mg/kg) and captopril (40 mg/kg) on body weight on Dex-induced hypertension. Values are means ± SEM for six rats. As compared to Dex control group, *: *P* < 0.05, **: *P* < 0.01, and ***: *P* < 0.001. As compared to saline control group, ^#^: *P* < 0.05, ^##^: *P* < 0.01, and ^###^: *P* < 0.001.

The body weight significantly decreased in Dex-induced hypertensive rats when compared to saline control group (*P* < 0.001). These changes were modified by LF administration (300 mg/kg) but not by captopril (Figure 3).

### 3.3. Effect of Lactoferrin on Thymus Weight

**Figure 4:**
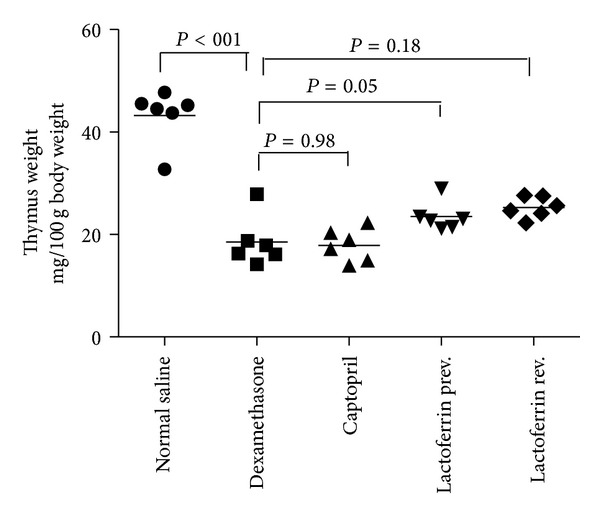
Effects of oral lactoferrin (300 mg/kg) and captopril (40 mg/kg) on thymus weight on Dex-induced hypertension. Values are means for six rats.

Dex significantly decreased thymus weight (*P* < 0.001) but LF and captopril had no effect on it (Figure 4).

### 3.4. Effect of Lactoferrin on Plasma H_2_O_2_ Concentration

**Figure 5:**
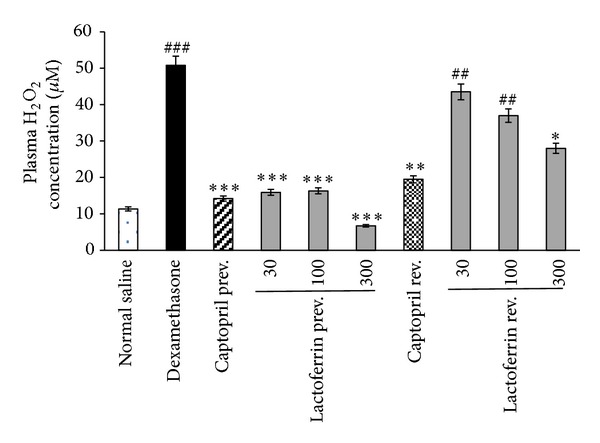
Effects of oral lactoferrin (30–300 mg/kg) and captopril (40 mg/kg) on plasma H_2_O_2_ concentration on Dex-induced hypertension in prevention (Prev) and reversal (Rev) groups. Values are means ± SEM for six rats. As compared to Dex control group, *: *P* < 0.05, **: *P* < 0.01 and ***: *P* < 0.001. As compared to saline control group, ^##^: *P* < 0.01, ^###^: *P* < 0.001.

Dex treatment significantly raised the level of plasma H_2_O_2_when compared with saline control rats (*P* < 0.001). LF administration significantly (*P* < 0.001) prevented the rise in H_2_O_2_ concentration at all doses in prevention study and reduced the elevated plasma H_2_O_2_ concentration at dose of 300 mg/kg in reversal study (*P* < 0.05). Administration of captopril also prevented and reversed the elevated plasma H_2_O_2_ concentration in Dex-induced hypertensive rats (Figure 5).

### 3.5. Effect of Lactoferrin on FRAP Assay

**Figure 6:**
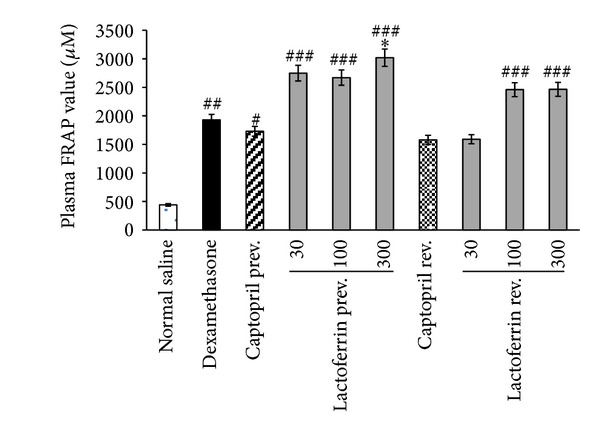
Effects of oral lactoferrin (30–300 mg/kg) and captopril (40 mg/kg) on plasma FRAP value on Dex-induced hypertension in prevention (prev.) and reversal (rev.) groups. Values are means ± SEM for six rats. As compared to Dex control group, *: *P* < 0.05. As compared to saline control group, ^#^: *P* < 0.05, ^##^: *P* < 0.01, and ^###^: *P* < 0.001.

Dex-induced hypertensive rats exhibited significant increase in the plasma FRAP values compared with saline control rats (*P* < 0.001). LF administrations also significantly increased FRAP values in Dex-induced hypertensive group at all doses of prevention study (*P* < 0.001) and at doses 100 and 300 mg/kg of reversal study (*P* < 0.05). In the captopril prevention study, FRAP values were also significantly increased (Figure 6).

## 4. Discussion

The results of this study showed antihypertensive effect of LF in Dex-induced hypertension. LF treatment dose dependently prevented and reversed a rise in SBP upon Dex administration. These LF effects were obtained in spite of the fact that dose of Dex used in the current study was 3-fold higher than that of dose used in most studies (10 *μ*g/kg/day) [8].

The exact mechanism of Dex-induced hypertension has not been fully understood. The role of augmented vascular pressor responsiveness, increased vasoconstrictor system activity including renin-angiotensin, endothelin, and sympathetic system, deficiency in vasodilators such as NO and prostanoids, hemodynamic alterations, and oxidative stress have been proposed as the mechanisms of Dex in induction of elevated blood pressure [26]. Structural, functional, and mechanical disruption in vessels have important role in Dex hypertensive effect. Dex results in increased sensitivity of vascular smooth muscles to vasoconstrictors and also increased vasoconstrictor release including angiotensin II, endothelin, and catecholamines [26, 27].

NO deficiency occurs during Dex treatment by downregulation of endothelial NO synthase expression and also through increased NO removal by oxidative stress [28]. Dex enhances superoxide free radical production through NAPDH oxidase pathway in vasculature when given chronically. It also causes significant alterations in antioxidant status [4]. Some antioxidants such as tempol, apocynin, and N-acetylcysteine have been shown to prevent Dex-induced hypertension [6, 26, 29].

LF as a food-derived peptide is believed to be safer than the drugs currently used for hypertension treatment [30]. Previous studies have reported blood pressure lowering effects of LF [19–21]. LF may cross and be internalized via a specific low density lipoprotein receptor-related protein in some capillary endothelial cells and it acts as an antioxidant factor [10]. The antihypertensive effects of LF may be attributed to the ACE and ECE inhibitor action and also endothelium-dependent relaxant action of LF [19–21]. It has been suggested that the vasodilatory action of LF is strongly mediated by NO production because of complete blockade of this effect by a NO synthase inhibitor [19]. Ikeda and coworkers have reported significant phosphorylation of Src, Akt, and endothelial nitric oxide synthase (eNOS) after treatment with LF suggesting a Src Akt eNOS-dependent pathway in promotion of vascular endothelial cell function by LF [31].

Furthermore, our results exhibited antioxidant effect of LF in Dex-induced hypertension. Hypertension of Dex was accompanied by increased plasma hydrogen peroxide level. LF administration dose dependently prevented and reversed H_2_O_2_ overproduction during Dex injection. Our finding showed significant increases in plasma ferric reducing antioxidant power upon Dex administration. It has been reported that glucocorticoids may activate the antioxidant enzymes in some tissues due to the state of oxidative stress and in a tissue specific manner [4]. However, the antioxidant capacity was higher in LF pretreated rats than that of rats treated with Dex alone. The potent antioxidant action of LF has been reported in other studies. Two weeks LF supplementation has been able to increase the hydrophilic antioxidant capacity in healthy humans [14]. LF has protective effect on pathological circumstances associated with iron-catalysed ROS based on its metal ions-binding capacity [32]. It has been suggested that LF contributes in oxidoreductive reactions at the cell membrane. An antioxidant effect of LF on erythrocytes through inhibition of lipid peroxidation and hemolysis has been reported [15]. LF is an important specialized iron scavenger and its antioxidant activity is most likely linked to its ability to bind ferrous and ferric ions. Thus, LF may inhibit the iron-catalyzed formation of hydroxyl radicals through Fenton reaction—an important source of ROS [11, 33]. Overproduction of ROS and their interaction with NO may contribute to reduction of NO bioavailability and therefore antioxidant effect of LF would prevent NO deficiency.

The result of this investigation suggested the antioxidant effect as another mechanism responsible for antihypertensive effects of LF. Our results showed effectiveness of LF in both prevention and reversal studies. Although some antioxidants such as tempol have been effective only in the prevention study LF with potent antioxidant effect could normalize the SBP even in Dex-induced hypertensive rats [6].

Metabolic consequences such as muscle and fat catabolism result in decreasing the rate of body weight gain during Dex administration. LF could prevent the body weight loss effect of Dex. Wakabayashi et al. have also reported protective effect of LF in preventing weight loss during infection with herpes simplex virus type 1 in mice. This effect of LF may be mediated through immune modulation and inhibition of proinflammatory cytokines production [34].

In conclusion, chronic LF treatment strongly reduced production of ROS and improved antioxidant capacity and reduced SBP in Dex-induced hypertension. These findings confirm the role of oxidative stress in the pathogenesis of Dex-induced hypertension and provide evidence that antioxidant effect may play a role in the antihypertensive effect of LF. With regard to high antihypertensive activity with safety, LF could be suggested for clinical trial studies for prevention and/or treatment of hypertension.
